# Bisphenols and their role in female infertility and hormone-related cancer

**DOI:** 10.1007/s12020-025-04521-3

**Published:** 2026-01-06

**Authors:** Marta Justyna Kozieł-Leszczyńska, Agnieszka Wanda Piastowska-Ciesielska

**Affiliations:** 1 Department of Cell Culture and Genomic Analysis, Medical University of Lodz, Lodz, Poland; 2 BRaIn Laboratories, Medical University of Lodz, Lodz, Poland

**Keywords:** Bisphenols, Infertility, Breast cancer, Ovarian cancer, Endometrial cancer

## Abstract

Various types of external chemicals can disrupt the endocrine system, interfering with normal hormone function and causing a broad spectrum of negative health effects. Endocrine-disrupting chemicals (EDCs) are a diverse group of natural and synthetic chemicals that are known to contaminate the environment. It is postulated that these agents can contribute to the development of many diseases, including infertility and cancer, because of their ability to interfere with estrogen receptors (ERs). Bisphenols (BPs) are a group of compounds that belong to EDCs, the most common of which is bisphenol A (BPA). Due to restrictions on the use of BPA in industry, analogues such as bisphenol S (BPS) and bisphenol F (BPF) have been introduced. However, some reports indicate that BPA analogues also have negative effects on the endocrine system in both humans and animals because of their structural similarity. This review summarises current knowledge related to BPA, its analogues and their role in female infertility and hormone-related cancers. Furthermore, this review also points to the problem of exposure to more than one estrogenic agent and highlights the importance of considering exposure to multiple chemicals when assessing health effects and setting daily limits.

## Introduction

**Fig. 1:**
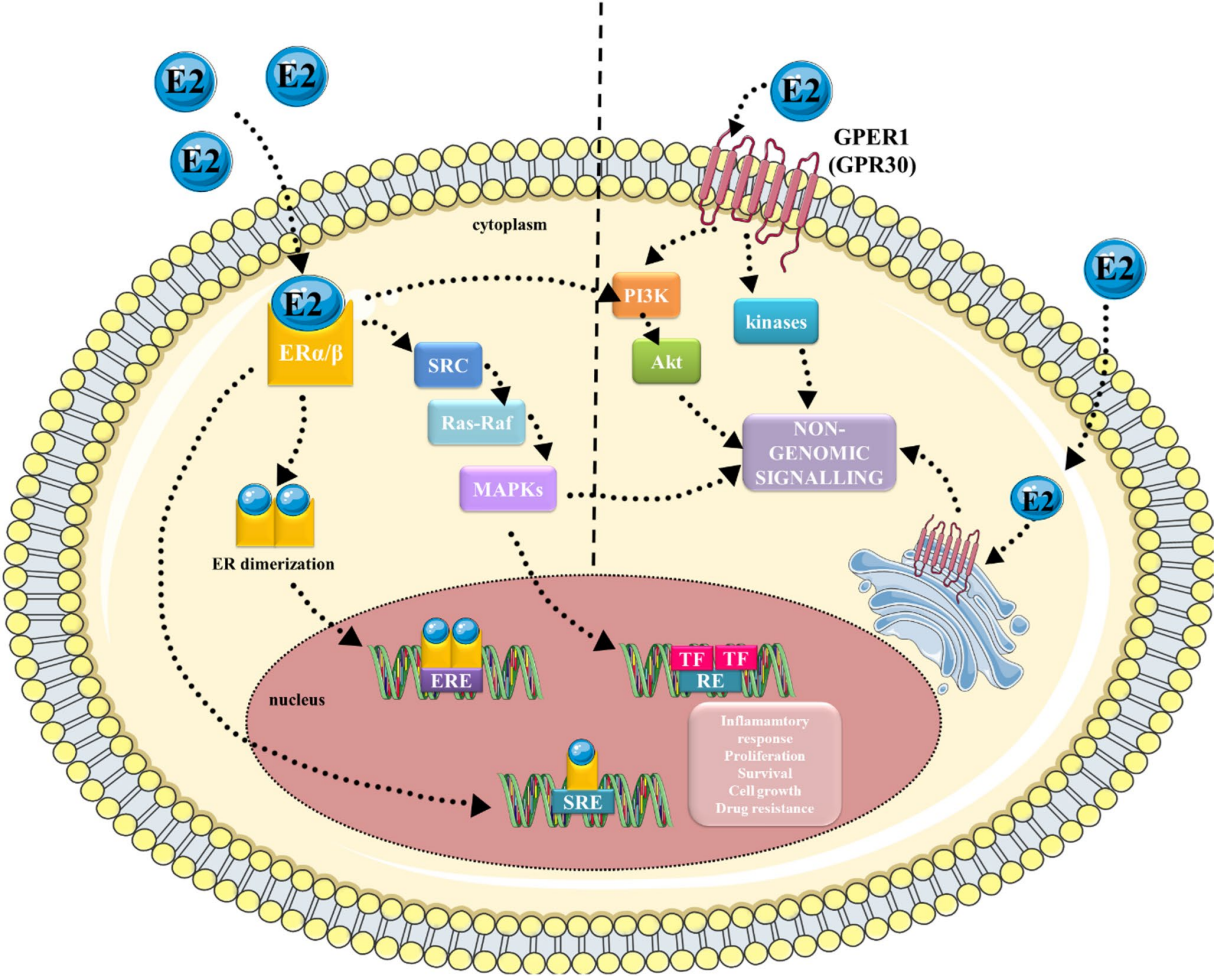
Genomic and non-genomic in estrogen signalling. In genomic signalling, estrogen binds to the ERα and/or ERβ in the nucleus. ERs dimerise and transport into the nucleus, where they bind with EREs or SREs. There, they cause activation or repression of the transcription of target genes. Non-genomic signalling acts through activation of kinase cascades in the cytoplasm or interaction with growth factor receptors in the plasma membrane. E2- 17β-estradiol, ERα- estrogen receptor α, ERβ- estrogen receptor β, GPER1- G protein-coupled estrogen receptor 1, ERE- estrogen response elements, SRE- steroid response element, TF- transcription factors, RE- response elements, SRC- Steroid Receptor Coactivator, Ras-Raf- renin-angiotensin system- residual adversarial fusion, MAPKs- mitogen-activated protein kinases, PI3K- phosphoinositide 3-kinase, Akt- protein kinase B. The graphical illustration was prepared using images from Servier Medical Art by Servier. Minor modifications were made (e.g., the colour of the stock images and some shapes) (https://smart.servier.com/smart_image/)

Endocrine-disrupting chemicals (EDCs) are a varied group of natural and synthetic chemical compounds defined as “exogenous compounds or mixtures that alter the function (s) of the endocrine system and consequently cause adverse effects in an intact organism, its progeny, or (sub) populations” [1]. Their significant effect on human health has been highlighted over the last years, mainly due to their common presence in the environment. Recent reports indicate that EDCs are present in almost all products used in everyday life, such as plastic bottles, detergents, food, cosmetics, and pesticides [2]. Moreover, it was also presented that bisphenol A (BPA) and its modified forms may be released from resin-based dental composites [3]. In general, transmission to the organism could occur via ingestion, dermal exposure, and inhalation, whereas ingestion is the most recent way of exposure [2]. Most of them have been reported to be present in human fluids such as milk, amniotic fluid, serum, urine, blood, saliva, and even adipose tissue [4–7]. EDCs are considered harmful to humans and animals, as well as to their welfare. They are reported to disorganise hormonal pathways due to interference with hormone biosynthesis, signalling, and metabolism [8, 9]. Many EDCs exhibit estrogenic activity and disrupt normal estrogen signalling, which is transmitted through estrogen receptors [10]. EDCs that affect estrogen receptors (ERs) signalling can modify both genomic and non-genomic ER activity through direct interactions with the ER or indirectly through transcription factors such as the aryl hydrocarbon receptor (AhR), or by modulating metabolic enzymes that are critical for normal estrogen biosynthesis and metabolism [8] (Fig. 1). In classical genomic signalling, estrogen (usually 17β-estradiol) binds to ERs. The receptors dimerise and bind to estrogen response elements (EREs) or steroid receptor response elements (SREs) in the nucleus [11]. They activate or repress the transcription of the target gene. Sometimes, ERs do not bind to EREs [11]. Instead, they engage in protein-protein interactions with other transcription factors that indirectly influence gene expression [11]. In turn, the estrogen receptor 1 (GPER1) is located on the cell membrane, triggering rapid responses (Fig. 1) [12]. However, even though BPs are reported to have estrogenic and anti-estrogenic activity, it is worth highlighting that many studies have shown that they may also have androgenic and anti-androgenic effects in cells [13]. As a consequence of hormonal imbalance, EDCs can cause many diseases, including, for example, infertility and hormone-dependent cancers [14–17]. Furthermore, EDCs have been reported to affect the development of the foetus, especially when the organs and neural system are formed [10, 15, 18], as well as affecting steroid pathways that could result in obesity, diabetes mellitus, and cardiovascular and reproductive disorders [19]. Although several studies confirm the link between exposure to EDCs and population diseases, the direct cause-and-effect relationship in many cases has not yet been revealed. Therefore, EDCs and their effect on human health are still being investigated. Exposure to EDCs has been widely described to be related to the formation and development of cancer arising from these tissues [20–22]. Due to the vast number of articles showing the effects of bisphenols (BPs), a group of compounds belonging to EDCs, on hormone-dependent cells, we have decided to summarise the knowledge and provide a general overview concerning the effects of BPA, its analogues, but also to present the possible synergistic effect of these compounds with other compounds that pollute the environment.

## BPA and its analogues

BPs are a group of chemical compounds that belong to EDCs, from which BPA is the most common and researched compound [23]. Humans are exposed to BPA and its substitutes mainly through food, but also through ingestion of dust and percutaneous absorption from thermal paper [24]. Interestingly, it was presented that BPA and BPA derivatives can also be released from resin-based dental composites and adhesives and thus also enter the human body [3, 25]. The key factor in the release of these compounds is the incorrect use (such as improper light curing) and storage of resin-matrix composites [3, 26]. Although most studies have shown that the local and systemic toxicity of the released compounds is relatively low, it should be emphasized that the studies are inconsistent, and this is not the only route of exposure, and therefore, these compounds may interact with others, for example, those ingested through food, and induce different effects [26]. Therefore, further research is needed. Due to the emergence of BPA substitutes in the industry, it is highly possible that humans, animals and the environment are already exposed to them, and scientists indicate that they are not a safe alternative to the already banned BPA [24]. The tissues of the breast, ovary and endometrium are the most sensitive to hormonal disturbances in a woman’s body. BPA was first synthesised in 1905 by condensing phenol and acetone in the presence of acid [27]. It was used for the preparation of various compounds used in everyday life, starting from children’s toys, plastic bottles, thermal paper and food containers to other plastic products [28]. Generally, people are exposed to BPA through food products, but its presence is also detected in water, soil and air [29]. In 90% of urine samples obtained from the United States population, BPA was observed [30, 31]. A recent Horizon 2020 research initiative, HBM4EU, found that 92% of adult participants from 11 European countries had detectable levels of BPA in their urine, raising long-term health concerns for all [32, 33]. Many epidemiological studies showed that exposure to BPA is associated with hormone imbalance and is due to infertility [34, 35]. The safe dose established by the U.S. Environmental Protection Agency (EPA) for BPA is 1 µg/kg per day. However, a dose of 0.2 µg/kg affects fertility in mice [36]. In April 2023, the European Food Safety Agency (EFSA) changed the tolerable daily intake (TDI) of BPA from 4 µg/kg body weight to 0.2 ng/kg body weight [29, 37]. Such a drastic reduction in TDI only underscores how destructive it is to health. It was postulated that BPA possesses both estrogen-like and anti-androgen characteristics and therefore can alter hormonal balance and lead to numerous diseases associated with the endocrine system, such as infertility, metabolic disorders or hormone-dependent cancers (e.g. prostate, ovarian or breast cancer) [28]. Because in Europe BPA was banned in the production of some products such as baby bottles or infant formula packaging, its analogues, such as, for example, bisphenol S (BPS), bisphenol F (BPF), bisphenol AF (BPAF) and tetrabromobisphenol A (TBBPA), are increasingly being used in industry. In recent years, the application of BPA analogues has raised new concerns. First, due to the limited data on their toxicology for both human and animal health. Secondly, scientists have already suggested that these analogues work similarly to the basic form due to the high structural similarity between them [38]. In recent years, another healthcare issue has also been considered: the exposure of more than one compound with estrogenic activity and its possible synergistic action [39]. Most people in developed countries are regularly exposed to a variety of synthetic chemicals that disrupt the endocrine system. Studies from the last five years underline the necessity of evaluating the combined effects of EDCs rather than testing individual chemicals, due to the different responses. Zhan et al. concluded that co-exposure to BPA and diethylhexyl phthalate (DEHP) is significantly related to the risk of infertility [40]. Yange et al. showed that combined exposure to BPA, BPS and BPAF corresponds with type 2 Diabetes mellitus (T2DM) risk [41]. Pollock et al. demonstrated that a mixture of triclosan, tetrabromobisphenol A (TBBPA), butyl paraben, propyl paraben and di(2-ethylhexyl) phthalate increase BPA concentrations in the lungs, muscle, uterus, ovaries, kidney, and blood serum of female CF1 mice [42]. Reina-Pérez et al. suggested that the mixture of BPA, BPS and BPF stimulates adipogenic differentiation of human adipose-derived stem cells (hASCs) [43]. They also stated that the response of these cells is different when they are treated only with one compound [43]. In turn, genistein (GEN) was found to reduce adverse effects stimulated by BPA in rat offspring [44]. These results highlight the importance of considering exposure to multiple chemicals when assessing health effects and setting permissible exposure limits.

## Metabolism of BPs

**Fig. 2:**
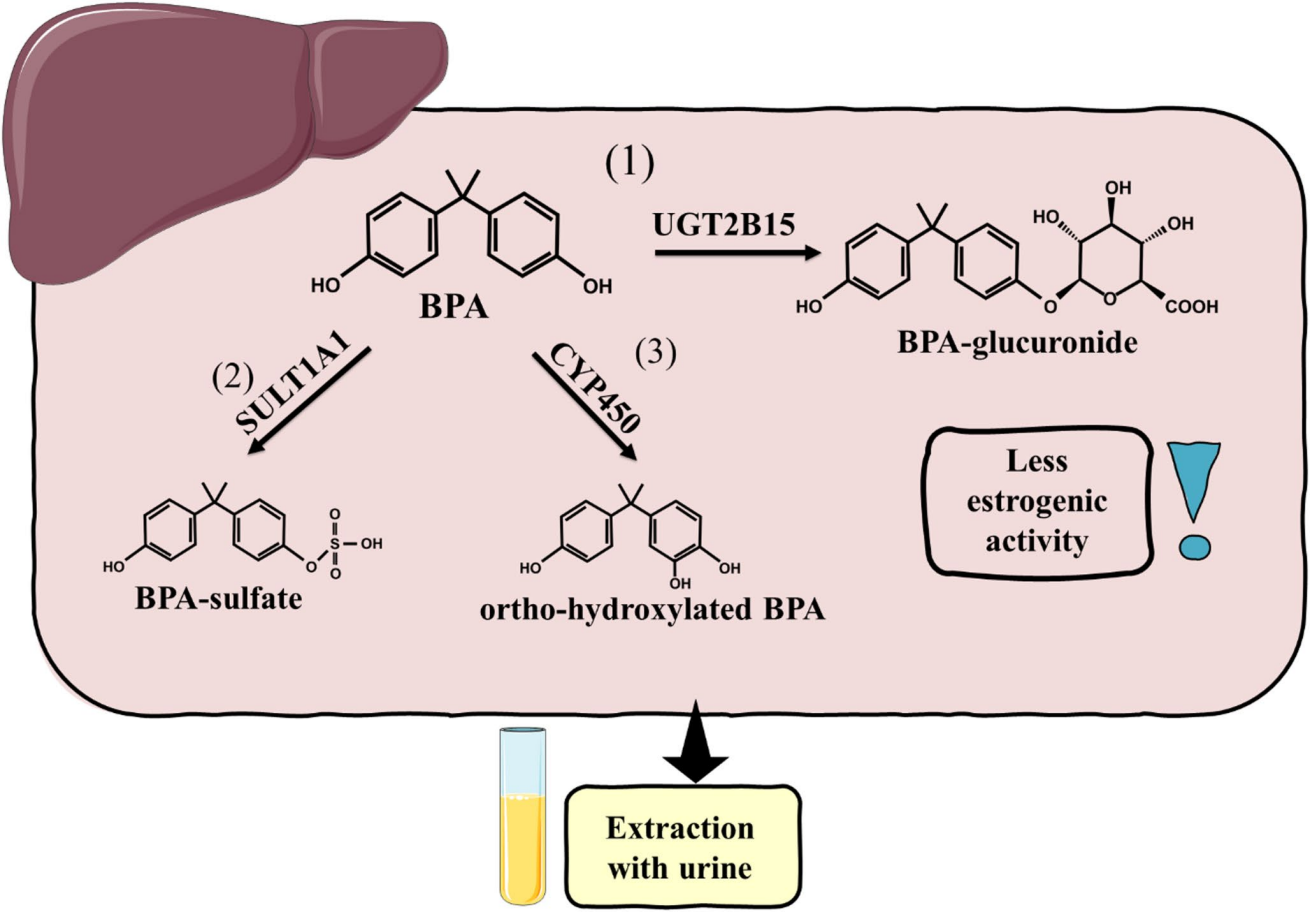
Metabolism of BPA in the liver. BPA is metabolised mainly by glucuronidation (1) but possibly also sulfation (2) or oxidation (3). Metabolization is followed by excretion mainly through urine. BPA- Bisphenol A, UGT2B15- UDP-glucuronosyltransferase 2B15, SULT1A1- Sulfotransferase 1A1, CYP450- Cytochrome P450. The graphical illustration was prepared using images from Servier Medical Art by Servier. Minor modifications were made (e.g., the colour of the stock images and some shapes) (https://smart.servier.com/smart_image/)

The main site where BPA biotransformation takes place is the liver, more precisely its endoplasmic reticulum [27]. Nevertheless, biotransformation of its analogues does not necessarily occur there. For example, it has been observed that the main site of BPF biotransformation is the intestine [27]. The biotransformation process generally involves glucuronidation, making BPs more soluble in water and excreted in urine [27]. The main enzymes involved in the metabolism process of BPs in the liver are 5’-diphosphate-glucuronosyltransferases (UGTs) 2B15 and 1A9 [27]. In breast tissue, for example, UGT1A1 was reported to be necessary [45]. In addition to glucuronidation, sulphation is also possible. However, BPA-sulphate and its analogous sulphates are observed in patient samples in much smaller amounts than the glucuronide form [27]. It was reported that sulfotransferase (SULT) 1A1 is the main enzyme responsible for BPA sulfation [27]. Other enzymes that can affect the structure of BPs belong to the cytochrome P450 (CYP450) family; of particular note here are those of the CYP1A and CYP2B subfamilies, as they are mainly responsible for the metabolism of BPA [27]. CYP-mediated oxidation leads to the production of stable hydroxylated compounds, including 4-methyl-2,4-bis (4-hydroxyphenyl) pent-1-ene, 5-hydroxy-BPA (5-OHBPA) and 3-hydroxy-BPA (3-OHBPA). In the case of BPAF, enzymes CYP3A4, CYP2C19 and CYP2C9 were found to be involved [46]. However, these changes can be associated with the generation of reactive species and thus lead to oxidative stress or DNA damage [46]. Nevertheless, BPs metabolism by CYP450 can vary according to genetic variant, age, and even gender, affecting faster or slower metabolism [47]. It should be noted that metabolic activity is limited in other tissues. Furthermore, BPA and some of its analogues have a high affinity for ERs and can therefore migrate to tissues where they are present [48]. All this contributes to the accumulation of this compound, such as, for example, the ovaries, breasts, or uterus. It is worth emphasising that co-exposure with other EDCs may disturb the normal metabolism of BPs. For example, it was proven that phthalates may modulate the activity of UGTs [49]. Soy isoflavones, GEN and daidzein were also reported to modulate phase II enzymes in Swiss Webster mice [50]. This suggests that our diet has a key influence on the metabolism and, therefore, on the exposure and accumulation of BPs in the organisms. A diagram showing the metabolism of BPA in the liver is shown in Fig. 2.

## Women’s reproductive health

In recent decades, environmental pollution caused by urbanisation and industrialisation has been found to affect human health, including fertility [51]. Infertility is estimated to affect 25% of couples in developing countries [51]. Proper cyclicity of changes in the ovaries and uterus (e.g. cycle duration, follicle maturation, ovulation and duration of menstrual bleeding) is necessary for pregnancy [52]. The reproductive cycle in both humans and animals seems to be one of the points that are disturbed by the action of BPs [52]. In in vivo studies, it was observed that BPA can disrupt ovarian follicle development and that this effect is associated with abnormal luteinizing hormone (LH) release [53]. The hypothalamic-pituitary-gonadal (HPG) axis plays a key role in female fertility, regulating the menstrual cycle and ovulation. Xi et al. showed that BPA increased the expression level of *kisspeptin-1* (*KiSS-1)*, *gonadotropin-releasing hormone* (*GnRH)* and *follicle-stimulating hormone* (*FSH)* in mouse pups, highlighting the possible effects of BPA on local hypothalamic and pituitary regulatory circuits [54]. Chen et al. showed that BPA, BPF and BPAF exposure altered mRNA expression of genes associated with the HPG axis (e.g. *CYP19b*, *17βHSD*, *3βHSD*, and *FSHR*) in marine medaka (*Oryzias melastigma*) [55]. Anselmo et al. demonstrated that a mixture of BPS and another EDC, tributyltin, increased *GnRH* and decreased *FSH* gene expression [56]. Furthermore, treatment with BPS alone or in combination with tributyltin also decreased serum FSH and LH levels in rats [56]. Decreased FSH levels may, in turn, be associated with a reduced ovarian stimulation, a greater number of atretic follicles and impaired follicle development observed in this study [56]. Overall, EDCs alone or in mixtures may contribute to dysregulation within the HPG axis, potentially impacting hormonal balance and thus leading to hormone-dependent disorders, including infertility. One of the diseases associated with infertility is primary ovarian insufficiency (POI), which results from the exhaustion of primordial follicles. BPA was postulated to modulate the transition from primordial follicles to primary follicles and consequently result in premature exhaustion [57]. Hu et al. postulated that this effect is associated with the deleted phosphatase and tensin homolog on chromosome ten (PTEN) signalling pathway [57]. BPA is also capable of influencing the development of antral follicles. Pertez et al. observed that BPA induces atresia and inhibits follicle growth [58]. On the molecular level, it was related to the modulation of genes associated with cell cycle regulation and apoptosis, i.e. *Cyclin-dependent kinase 4* (*Cdk4)*, *Cyclin E1* (*Ccne1)*, *transformation-related protein 53* (*Trp53)*,* Cyclin D2 (Ccnd2)*,* BAX* and *BCL2* [58]. Patel et al. observed that BPA modulates the number of follicles in rats and the concentration of sex steroid hormones [59]. It appears to be consistent with another study in which BPA also modulated hormone production and enzymes associated with steroidogenesis and altered antral follicles growth [60]. Ziv-Gal et al. showed that low doses of BPA reduce follicle growth partially via the AhR pathway [61]. Interestingly, researchers noted that exposure to BPA was correlated with a shorter luteal phase in female samples [62]. Tang et al. presented that chronic exposure to BPA leads to prolonged dioestrus and reduced ovulation in adult female mice [63]. In the case of BPA analogues, it was observed that the exposition of ewes to BPS resulted in a disturbed hormone ratio in both follicular and oviduct fluids [64]. Furthermore, BPS also affects the concentration of progesterone, estradiol, and estrone in plasma samples [64]. However, Huang et al. showed that the toxicity of BPA and its analogues is different [65]. They showed that BPA, BPS, BPF and BPAF affect the viability of KGN cells (human granulosa-like tumour cells), while the highest toxicity was observed after treatment with BPAF [65]. Furthermore, reactive oxygen species (ROS) concentration after BPA treatment in these cells was also elevated, and, at the same time, antioxidant capacity was decreased [65]. BPS induces oxidative stress in ovarian tissue and disrupts hormonal balance (LH and FSH) [66]. Prenatal exposure of mice to BPA resulted in abnormal estrous cyclicity, disturbed follicular development, fertility disturbances manifested by lower pregnancy rate, problems with parturition and elevated levels of dead pups, increased levels of testosterone and dysregulated expression of steroidogenic enzymes in the ovary [67]. The interruption after treatment with BPS was also observed *in vitro* [68]. In ewes, BPS affects the quality and secretion of progesterone of the corpus luteum [69], estradiol concentrations, and oocyte development [64, 70]. Interestingly, it was postulated that BPS may have an even greater effect, however, after fertilization [71]. BPA and BPS damage ovarian granulosa cells, but through the activation of different, as yet unknown molecular pathways [72, 73]. Huang et al. noticed that co-exposure of BPA and BPAF induces ROS production and apoptosis in KGN cells [74].

## Polycystic ovary syndrome (PCOS)

Polycystic ovary syndrome (PCOS) is a disease that affects women of reproductive age and is one of the most common causes of infertility [75]. Moreover, despite fertility problems, it is also associated with obesity, metabolic syndrome, diabetes, endometrial hyperplasia, and even the cardiovascular system [76]. The main symptoms observed in PCOS are menstrual irregularities and hyperandrogenism [76]. In ultrasound images, the ovaries have many small follicles that have stopped maturing, most often due to hormonal disturbances in the body [77]. The main cause of this disease is not yet well understood, but prolonged exposure to EDCs may have an impact on the development of this syndrome [78]. However, the aetiology of PCOS remains unclear, and thus, more studies should be performed to clarify the possible association between EDC and the occurrence of this syndrome. Although BPA seems to be the best-known compound of EDCs, its role in the emergence of PCOS is still unclear. Some case-control studies tried to verify whether there is an association between BPA concentration in human fluids and PCOS. Many studies reported that the concentration of BPA in serum is significantly higher in PCOS patients than in healthy women [79–82]. Interestingly, opinions are divided when it comes to its analogues. There are studies that postulated that the concentration of BPA analogues does not differ between healthy and unhealthy patients [82], but on the other hand, there are also studies that demonstrated increased probability of PCOS in patients exposed to BPA and its analogues (BPAF, BPAP, BPB, BPP, BPS, and BPZ), especially in obese women [83]. This difference may be due to the population studied, as it is known that exposure in different parts of the world can vary [32]. As demonstrated by Tarantino et al., the liver-spleen axis plays an important role in the pathogenesis of PCOS [84]. And as it turned out, BPA is also involved in the disruption of this axis [84]. They showed that concentration of BPA is associated with insulin resistance, inflammation and elevated androgen levels [84]. It seems that BPA, through the induction of oxidative stress, leads to enlargement of the spleen and thus disrupts the liver-spleen axis [84]. Kandaraki et al. suggested that BPA may influence androgen metabolism and insulin action and thus participate in the pathophysiology of PCOS [79]. Research from Poland also showed that BPA levels positively correlated with testosterone levels and free androgen index (FAI) [80]. However, they did not observe significant disturbances in insulin concentration [80]. On the other hand, Jurewicz et al. did not observe a correlation between BPs concentration and metabolic parameters such as insulin [82]. In Turkey, PCOS-adolescent girls also had higher concentrations of BPA in serum, and similarly to other studies, BPA concentration was significantly correlated with androgen levels [81]. Zhou et al. suggested that BPA may reduce ovarian reserve, an oocyte number, in PCOS patients and that BPA was negatively correlated with antimullerian hormone (AMH) [85]. The concentration of BPA was also investigated in urine samples, and the trend was similar to that in serum [86–88]. In addition to case-control studies, animal models were also used. Markey et al. presented that prenatal exposure to BPA in mice resulted in altered morphology and function of the ovaries [89]. In the *in vivo* model, a decreased level of LH was also observed in plasma [90]. Numerous studies showed that BPA administered during the neonatal period of rats induces changes in the morphology of the ovaries (multiple cysts and changes in the area occupied by the corpora lutea) and disturbances in the oestrous cycle [91–93]. Moreover, Fernández et al. concluded that BPA alters the GnRH signalling and affects the HPG axis and thus contributes to PCOS [93–95]. Interestingly, BPA was also shown to alter the release of thyroid-stimulating hormone (TSH) [95], suggesting that BPA alters all necessary pathways involved in the PCOS pathophysiology. BPA metabolism also plays a crucial role in PCOS. Some differences in BPA concentration have been shown between the sexes [96]. In men, the level of BPA was significantly higher than in the group of women with PCOS, compared to the group of healthy women [97]. The authors hypothesised that this difference may be associated with androgen-related metabolism [97]. Then, Takeuchi et al. proposed that this difference is due to the different expression of UDP-glucuronosyltransferase 2B1 (UGT2B1) between genders [96]. Interestingly, high levels of androgens decrease the activity of liver enzymes, which may result in impaired metabolism of BPs [98]. These findings raise interest in whether BPA is an inducer of PCOS, or whether its accumulation in fluids from patients with PCOS is the result of a disturbed metabolism. However, to date, no unequivocal position has been taken on this matter.

## Endometriosis

Endometriosis (EM) is another gynaecological disease that is associated with infertility in women and belongs to chronic inflammatory diseases [99]. EM is one of the least understood diseases in the field of gynaecology [100]. EM occurs when the cells that normally line the uterus are present outside the uterine cavity, creating lesions on the ovaries, intestines, bladder and many other organs [101]. To this date, the cause of this disease is not clear; therefore, prevention, diagnosis and treatment are extremely hard. Some reports have reported that EM may be related to extensive exposure to EDCs, and because its aetiology remains unknown, this issue should be thoroughly evaluated [102]. The relationship between BPA exposure and the incidence of endometriosis is unclear. Some authors have described the association between BPA concentration and endometriosis [103–105], while other authors showed that this association does not exist [106–108]. Cobellis et al. observed the possibility of a link between serum BPA levels and endometriosis [104]. They observed that the presence of at least one of the two BPs tested (BPA and bisphenol B (BPB)) was confirmed at a level of up to 63.8% in the serum of women with endometriosis, suggesting the relationship between this disease and exposure to BPs [104]. The results carried out by Simonelli et al. are in line with those results, confirming them [103, 104]. Peinado et al. showed that exposure to BPA is associated with endometriosis but did not see this association for BPS and BPF [105]. Forte et al. investigated the effects of BPA on human endometrial stromal cells (ESCs), derived from endometrial biopsies from healthy women [109]. It has been observed that exposure of cells to BPA causes G2/M cell cycle arrest and increased cell migration [109]. Furthermore, it also affected the expression of *insulin growth factor binding protein 1 (IGFBP1)* and *prolactin (PRL)*, thus enhancing the effect of progesterone [109]. The authors also reported changes in the expression of matrix metalloproteinase-3 (MMP-3), matrix metalloproteinase-9 (MMP-9) and metalloproteinase inhibitor 3 (TIMP3) [109]. These data demonstrated that BPA could alter several features of the human endometrial physiology and thus contribute to the endometriosis-like phenotype [109]. Wen et al. showed that the concentration of BPA, in serum obtained from patients was positively correlated with the levels of matrix metalloproteinase 2 (MMP-2) and matrix metallopeptidase 9 (MMP-9) and with the risk of endometriosis [110]. It is worth remembering that these metalloproteinases are used in cell evaluation of the invasion and that they are known to be crucial in the development and progression [111]. Furthermore, it was also shown that in an *in vitro* endometriosis model, BPA increased the expression of these metalloproteinases in a dose-dependent manner, and doses of 10 nM and 100 nM increased the invasive nature of human embryonic stem cells (HESCs) [110]. Interestingly, this effect was also noticed to be abolished by blocking GPER1 or mitogen-activated protein kinase (MAPK)/ extracellular signal-regulated kinase (ERK) signalling pathway [110]. Xue et al. presented that BPA stimulates the development of endometriosis by damaging the balance between ERα and ERβ and thus stimulates the expression of ERβ pro-proliferative abilities, via targeting GPER1/Phosphoinositide 3-kinase (PI3K)/ mammalian target of rapamycin kinase (mTOR) and WD repeat-containing protein 5 (WDR5)/ Tet methylcytosine dioxygenase 2 (TET2) pathways [112]. Prenatal exposure to BPA resulted in endometriosis-like structures in treated mice [113]. In *in vivo* model, Kendziorski et al. aimed to evaluate the role of BPA in the pathogenesis of endometriosis in two female adult mouse strains (C57Bl/6 N and CD-1 mice) [114]. Mice were exposed to dietary BPA or 17α-ethinyl estradiol, as a positive control, for 12–15 weeks [114]. In both strains, an increase in endometrial gland nest formation and accumulation of stromal and periglandular collagen were observed [114]. BPA also led to increased levels of collagen type I alpha 1 chain (Col1a1) and collagen type III alpha 1 chain (Col3a1) expression and decreased expression of MMP-2 and matrix metallopeptidase 14 (MMP-14) [114]. All these changes described by the authors are associated with BPA and contribute to an endometriosis-like phenotype [114]. The results also showed that these two strains have different sensitivity to BPA (C57Bl/6 N > CD-1), suggesting that sensitivity to BPA also depends on genetics [114]. In pregnant mice (BALB/C strain), low doses of BPA result in increase of endometriosis-like structures in the adipose tissue which surrounds genital organs [113]. In addition, cystic ovaries, adenomatous hyperplasia, with cystic endometrial hyperplasia and atypical hyperplasia were observed [113]. Postnatal exposure to low doses of BPA in CD-1 mice also led to increased cystic ovaries and cystic endometrial hyperplasia [115].

## Bisphenols and cancer risk and development

Extensive research over the past decade has shown a close link between BPA exposure and the occurrence of various cancers, in particular breast and ovarian cancer, as well as endometrial cancer [116, 117]. However, the impact of BPA analogues and co-exposure with other xenoestrogens is still poorly understood. In this section, we will gather information on the effects of BPA and its analogues on breast, ovarian and endometrial cancer.

### Breast cancer

Breast cancer (BC) is the most common type of cancer that occurs among women [118]. BC is associated with environmental contaminants, such as BPA, that possess the ability to affect hormonal balance [116]. Research data on DNA damage, triggered by BPA, showed that this effect depends on ER in breast cancer cell lines [119]. Vivacqua et al. indicated that BPA acts as an agonist for ERα [120]. Lee et al. showed that BPA binds both to the ERα and ERβ and then increases proliferation via cyclin-dependent kinase 1/ cyclin-dependent kinase 2 (CDK1/2) and p38 MAPK kinase activation [121]. It was also shown that BPA disturbs cell cycle progression by affecting securin (PTTG1) and then miR-381-3p expression [122]. In turn, Nair et al. underlined that prolonged exposure to low doses of BPA for two months significantly increased their invasiveness and affected proteins that are associated with most oncogenic pathways [123]. More precisely, Lee et al. showed that BPA induces MCF7 cells proliferation by up-regulation of genes that participate in the control of cell cycle progression [124]. Zhang et al. showed that BPA in low doses increased migration and invasion of triple-negative cell lines (MDA-MB-231 and BT-549 cells) [125]. They observed that BPA modulates the expression of MMP-2 and MMP-9 both on gene and protein levels [125] and that this effect is surprisingly not mediated by GPER1, but by estrogen-related receptor γ (ERRγ) and ERK1/2 and protein kinase B (Akt) pathway [125]. Then, a similar relationship was observed in other breast cancer cell lines- MCF7 and SkBr3, where authors concluded that stimulated proliferation is not associated with ERα and GPER1 activation [126]. On the contrary, Xu et al. showed that BPA acts via the GPER1/ Caveolin-1 (Cav-1)/ HSP9 cascade in a hypoxic microenvironment [127]. Sanchez and colleagues and Pupo et al. also observed that BPA-induced migration is associated with GPER signalling [128, 129]. Furthermore, Dong et al. showed that BPA prompts activation via GPER1 [130]. Therefore, it seems that the role of GPER1 in response to BPA in breast cancer cells is not clear to date. Ansari and colleagues observed that MCF7 cells treated with BPA for approximately 200 days change their morphology [131]. Moreover, they also observed that the levels of mesenchymal markers changed [131]. Song et al. proposed that BPA induces migration of cells via modulation of inflammation [132]. Interestingly, low doses were also shown to affect response to chemotherapy. Huang et al. showed that BPA decreased the effectiveness of tamoxifen in MCF7 cells but not in MDA-MB-231 cells [133]. As mentioned earlier, concerns have also been raised about BPA analogues in recent years. The main reason is their commonality in ‘BPA-free’ products and no restrictions on use. However, also because of the fact that their toxicity remains underexplored. For example, BPS and BPF, replacements for BPA, are also hormonally active and may affect hormonal balance in organisms [134]. Huang et al. reported that BPS induces epigenetic changes in transposons in MCF7 cells and that this effect may be associated with the development of breast cancer [135]. Lin et al. observed that BPS stimulates proliferation and cell cycle progression only in ERα-positive cells and that this effect is mediated by the ERα-cyclin D-CDK4/6-pRb pathway [136]. In turn, Deng and colleagues reported that BPS induces migration of TNCB cells (MDA-MB-231) in a concentration range of 10-1000 nM; moreover, they also summarised that this effect is mediated by GPER1/Hippo-YAP signalling [137]. Kim et al. showed that BPA, BPS and BPF induce the migration and proliferation of MCF7 CV cells [138]. All bisphenols tested altered cell cycle progression by affecting cyclin D1 and cyclin E1 and the epithelial-mesenchymal transition (EMT) via *E-cadherin* and *N-cadherin* [138]. Lei et al. also showed that BPF induces MCF7 cells proliferation and that this effect is associated with increased production of ROS production [139]. Furthermore, they also reported that BPF may activate PI3K/PKB (phosphatidylinotidol 3-kinase/protein kinase B) and ERK 1/2 through GPER1 [139]. Furthermore, TCBPA was reported to act through PI3K/Akt and ERK 1/2 signalling, increasing the proliferation and migration of MCF7, MDA-MD-231, and SKBR3 cells [140]. Consistent with previous studies, Yu et al. also noted the effect of TCBPA on the PI3K/Akt signalling pathway but also indicated that it occurs through GPER1-EGFR [141]. The history of BPZ is little known. To our best knowledge, only Böckers et al. have shown that BPZ might affect genes that are linked to cell growth, invasion, migration, apoptosis and cancer development [142]. Del Favero et al. presented that co-exposition of BPA with estrogenic mycotoxins- alternariol (AOH) and α-zearalenol (α-ZEL), and fitoestrogen- GEN have an impact on the formation of the metastatic clones, affecting the cell cytoskeleton [143]. Moreover, they observed differential response after co-exposure with these compounds, suggesting the complexity of the interaction between these factors [143]. Taken together, it appears that the BPA analogues are as toxic as the original compound, and they may also have additional effects that we do not know about as a result of the still small amount of research done. Table 1 summarizes information presented in this section (Table 1).

**Table 1:** Detailed information about the effects of bisphenols on breast cancer cells *in vitro*

Substance	Cell line	Effect	Dose range used in experiments [nM]	Time of exposition	Bibliography
BPA	MDA-MB-231 BT-549	↑migration ↑invasion ↑*MMP-2* and *MMP-9*	1 × 10^1^	48 h	[125]
BPA	MCF7 SkBr3	↑proliferation ↓apoptosis ↑PCNA, Bcl-2	1 × 10^1^	48–96 h	[126]
BPA	MCF7	↑migration ↑invasion ↑N-cadherin, Vimentin, CD44, slug, α-SMA ↓E-cadherin	1 × 10^2^	Up to 200 days	[131]
BPA	MCF7	↑migration ↑DNA damage ↑invasion	0.0015– 0.0048	Two months	[123]
BPA	MCF7	↑proliferation	1 × 10^3^ – 10^4^	144 h	[124]
BPA	MDA-MB-231 SkBr3	↑proliferation ↑HIF-1α and VEGF	1 × 10^3^	24 h	[127]
BPA	MDA-MB-231	↑Migration ↑Invasion	1 × 10^3^	48 h	[129]
BPA	MCF7 CV	↑migration ↑proliferation	1.0 × 10^3^-1.0 × 10^4^	48–192 h	[138]
BPS	MCF7	↑proliferation	1 × 10^− 1^ – 1 × 10^5^	144 h	[136]
BPS	MDA-MB-231	↑migration	1 × 10^3^	48 h	[137]
BPS	MCF7 CV	↑migration ↑proliferation	1 × 10^3^–1 × 10^4^	48–192 h	[138]
BPF	MCF7 CV	↑migration ↑proliferation	1 × 10^2^–1 × 10^4^	48–192 h	[138]
BPF	MCF7	↑proliferation ↑ROS production ↑intracellular calcium (Ca2+) levels	1 × 10^1^ – 1 × 10^3^	24–72 h	[139]
TCBPA	MCF7 SKBR3 MDA-MB-231	↑proliferation ↑ROS production	1 × 10^1^ – 1 × 10^4^	24–72 h	[140]

BPA- bisphenol A, BPS- bisphenol S; BPF- bisphenol F; TCBPA- tetrabromobisphenol A; MMP-2- matrix metallopeptidase 2; MMP-9- matrix metallopeptidase 9; PCNA- proliferating cell nuclear antigen; Slug- zinc finger protein SNAI1; α-SMA- alpha-smooth muscle actin; HIF-1α- hypoxia-inducible factor 1-alpha; VEGF- vascular endothelial growth Factor; ROS- reactive oxygen species

### Endometrial cancer

In 2020, endometrial cancer (EC) was ranked as the sixth most common cancer in women worldwide [144]. Risk factors associated with the development of EC include a high number of non-ovulatory cycles, obesity, diabetes and hyperestrogenism [145]. The last may be related to increased exposure to exogenous estrogens [145]. At the molecular level, endometrial epithelial cell proliferation is strongly regulated by the estrogen and progesterone signalling pathways [145]. Therefore, disturbances in hormone-dependent signalling may contribute to the development of EC. Consequently, BPA and its analogues, which have been reported to have estrogenic activity, appear to have an effect on this type of cancer. Neff et al. observed that chronic exposure to BPA increases glandular epithelium [146]. It seems to be an interesting discovery, taking into account that glandular epithelium is the origin of endometrial hyperplasia and then cancer. Furthermore, BPA exposure was also linked with the abnormal weight of the uterus [146]. The authors stated that proliferation was stimulated via activation of the fibroblast growth factor receptor (FGFR) pathway and phosphorylation of the ERK1/2 and MAPKs [146]. In addition, BPA-targeted heart and neural crest derivatives expressed the 2 (HAND2) factor which is responsible for antiproliferative action [146]. In RL95-2 cells, BPA stimulates proliferation, migration and invasion via cyclooxygenase-2 (COX-2) [147]. Furthermore, prolonged exposure (14 days) is described to be associated with EMT [147]. Yaguchi presented that BPA is responsible for the nuclear translocation of ERRγ *in vitro* [148]. Interestingly, BPA also induced the influx of calcium ions [148]. Fan et al. showed that high doses of BPA, BPS and BPF (100 µM) decreased the viability of EC cells [149]. It was also presented that BPA, BPS and BPF downregulated ERα, ERβ and GPER1 proteins [149]. The authors also showed that high doses of BPA (10 and 100 µM ) and BPS (100 µM ) reduced the rate of spheroid attachment, and BPF did not disturb this process [149]. Interestingly, co-exposure of BPA with zearalenone (ZEN) was found to cause additive estrogenic effects in the Ishikawa cell line [39]. In contrast, a mixture of BPA and AOH expressed an antagonistic effect [39]. Table 2 summarizes information about the effects of bisphenols on endometrial cancer cells *in vitro.*

**Table 2:** Detailed information about the effects of bisphenols on endometrial cancer cells *in vitro*

Substance	Cell line	Effect	Dose range used in experiments [nM]	Time of exposition	Bibliography
BPA	RL95-2	↑Migration ↑Proliferation ↑Invasion	1 × 10^1^ – 1 × 10^4^	varies from experiment to experiment	[147]
BPA	HEC265 Ishikawa	↑Proliferation	1–1 × 10^3^	24 h	[148]
BPA BPS BPF	Ishikawa	↓Proliferation	1 × 10^5^	24–72 h	[149]

BPA- bisphenol A, BPS- bisphenol S, BPF- bisphenol F

### Ovarian cancer

Ovarian cancer is one of the most dangerous cancers due to its diagnosis at a very late stage. Each year, approximately 314,000 new cases and 207,000 deaths are observed worldwide [144]. Risk factors associated with an increased likelihood of ovarian cancer include age, diet and hormonal imbalance [150]. It was observed that BPA modulates the expression of genes involved in apoptosis and in the regulation of the cell cycle and therefore may destabilise and lead to apoptosis in ovarian cells [151]. Low doses of BPA stimulate the migration of ovarian cancer cells through upregulation of migration-related genes such as *MMP-2* or *MMP-9* [152]. Furthermore, Ptak et al. presented that MAPK and PI3K play a crucial role in response to BPA and that their blockade leads to the elimination of the promotory effect of BPA [152]. However, Park et al. also showed that BPA-induced proliferation is not fully related to MAPK pathway and mostly depends on ERs receptors [153]. Kim et al. noticed in BG-1 cells that the promotion of EMT was associated with estrogen signalling [154]. In line with the previous study, Sang et al. proposed that BPA interfere with migration and invasion in OVCAR3 cells as a result of ERα receptor activation [155]. It was observed that BPA can also stimulate migration and invasion in SKOV3 cells by inducing EMT, observed at the genomic level where *MMP-9* was up-regulated and *ZO-1* was downregulated [156]. ERα-positive cells were reported to be more sensitive to the action of BPA [157]. The WNT pathway was also reported to be involved in the response of cells to BPA [156]. Hayes et al. showed that BPA affects the expression of the *Sirtuin 1* (*SIRT1)* and H4-Lys-20 *histone-specific methyltransferase* (*SET8)* genes [158]. Ptak et al. observed that BPA modulates the expression of VEGF-R2 and VEGF-A in ovarian cancer cells but not in normal ovarian cells [159]. It has been clearly shown that BPA analogues also regulate the behaviour of ovarian cells [160, 161]. This suggests that they can also contribute to the development of ovarian diseases. Therefore, it seems necessary to assess the impact of these compounds on the health of both humans and animals. Especially in the context of cells that respond well to estrogen-like compounds. The mechanism of action of BPA analogues should be deeply evaluated. Table 3 presents the summarized information about the effects of BPA on ovarian cancer cells *in vitro.*

**Table 3:** Information about the effects of BPA on ovarian cancer cells *in vitro*

Experimental model	Effect	Dose range used in experiments	Time of exposition	Bibliography
OVCAR3	↑migration ↑MMP-2, MMP-9, CDH1	40–100 nM	24 h	[153]
OVCAR3	↑Migration ↑invasion	1–100 nM	120 h	[156]
SKOV3	↑Migration ↑invasion ↑MMP-9 ↓ZO-1	10–100 nM	24–72 h	[157]
BG-1	↑Migration ↑VIM, Snail, MMP-9	1000 nM	24–48 h	[155]
PEO1	↑Migration ↑Proliferation	1–1000 nM	24 h	[158]
OVCAR3 SKOV3	Modulation of *SIRT1* and *SET8*	10–50 nM	24 h	[159]
OVCAR3	↑Proliferation ↓caspase-3 activity	1–100 nM	72 h	[152]

MMP-2- matrix metallopeptidase 2; MMP-9- matrix metallopeptidase 9; CDH1- E-cadherin; ZO-1- Zonula occludens- 1; VIM- vimentin; Snail- zinc finger protein SNAI1; SIRT1- Sirtuin 1; SET8- histone H4K20-specific methyltransferase

## Conclusion

It is well known how BPA affects the endocrine system, hence its restriction on use in industry. However, the area concerning BPA analogues appears to be poorly described, which seems worrying given the reports indicating their negative effects on human and animal health. An in-depth understanding of the molecular pathways that are activated during exposure and taking into consideration co-exposure with other EDCs is essential to improve our awareness of the environmental pollutants present in our daily lives and, more importantly, will be a good basis for risk assessment.

## Data Availability

No datasets were generated or analysed during the current study.
